# Hormonal assessment of participants in a long distance walk

**DOI:** 10.1186/s13098-019-0414-1

**Published:** 2019-02-15

**Authors:** Haroldo Silva de Souza, Thiago Veiga Jardim, Weimar Kunz Sebba Barroso, Priscila Valverde de Oliveira Vitorino, Ana Luiza Lima Souza, Paulo César Veiga Jardim

**Affiliations:** 1 Hypertension League, Federal University of Goiás, 1a Avenida, S/N, Goiânia, GO CEP 74085-300 Brazil; 2 School of Social and Health Sciences, Pontifical Catholic University of Goiás, 76, 235 St., Goiânia, GO CEP 74175-120 Brazil

**Keywords:** Exercise, Hormonal, Glycemia, Thyroid hormones, Cortisol, Testosterone, Insulin

## Abstract

**Background:**

Exercise can disrupt homeostasis and trigger many adaptive responses in different hormonal axes. The study of hormonal interactions with physical activity is highly complex due to the number of variables, such as exercise duration, exercise intensity, individual level of training, circadian rhythm, nutritional status, and environmental conditions.

**Methods:**

This study was performed to assess daily variations of thyroid hormones, cortisol, testosterone, insulin and glucose during moderate to high intensity aerobic physical activity for 5 consecutive days. Sample collection was performed at baseline in the morning and in the evening, immediately after finishing the activity, on the 4 initial days of the activity. Statistical analysis was performed using software STATA V14. Continuous variables are presented as means and standard deviations, while categorical variables are presented as absolute and percentage values. We used Shapiro–Wilk, Wilcoxon Sign, Mann–Whitney and Student’s T test, according the needs.

**Results:**

The adrenocorticotropic axis showed an initial increase in the evening cortisol level compared to the baseline level in the morning (17.46 μg/dL and 15.97 μg/dL, respectively) and then exhibited a significant reduction between the 1st and 4th day of activity (17.46 μg/dL and 8.39 μg/dL, respectively; P = 0.001). The same pattern was observed for free thyroxine (T4) between the 1st and 4th day (1.31 and 1.14, respectively; P < 0.001).

**Conclusions:**

Moderate to intense long duration physical activity resulted in little variation in the hormones assessed, with a trend toward reduced levels of cortisol and free T4. These findings highlight an adaptive hormonal mechanism in response to stress that is repeated daily, as shown by cortisol and thyroid function in our study.

## Background

Exercise is the most potent “disrupter” of normal metabolism. Physical activity is a strong stimulus for the endocrine system, and the hormonal response to exercise is regulated by several factors, including intensity, individual level of training, duration, and type of exercise (endurance vs. resistance) [1].

Exercise, therefore, produces dramatic changes in homeostasis and the way in which it is tolerated and adapted is closely related to the hormonal regulation of physiological systems.

Most studies on the relationship between exercise and the endocrine system perform an initial and final (pre and post) assessment [2–5]. Few studies have analyzed the hormonal dynamics to evaluate the daily impact of repeated exercise for several consecutive days.

Studying these hormonal changes, particularly during walking, the most popular physical activity, may help better understand the hormonal homeostasis during this type of activity. This is especially important when walking is practiced outdoors for a long period of time, in a repeated manner, for several days. Such information will help better determine the ideal duration of training, rest, and time interval between training sessions. These data will, in turn, contribute to the improvement of the performance of athletes and to the optimization of exercise programs in non-athletes as a tool for health promotion and maintenance [6].

The aim of this work was to evaluate a hormone panel consisting of cortisol, testosterone, free T4 (FT4), total triiodothyronine (T3), thyrotropin (TSH), insulin, and glucose substrate during moderate to high intensity aerobic physical activity for 4 consecutive days to identify possible adaptive mechanisms of this hormonal environment in response to a daily stimulus.

## Methods

### Selection

The individuals studied were previously selected from a total of 110 candidates who voluntarily enrolled to participate in a walk. The group was chosen through a 2-day endurance test, where they covered 20% of the total walk (60 km) on the 1st day and 10% (30 km) on the 2nd day. Next, the candidates with the best times underwent clinical and laboratory assessments in a specialized center. All subjects were subjected to an ergometric test to evaluate cardiovascular risk.

A medical interview was conducted to collect the following variables: gender, marital status, age (years), body mass index (kg/m^2^), smoking history (yes/no), hypertension (yes/no), dyslipidemia (yes/no), thyroid disease (yes/no), diabetes (yes/no) and family history of heart disease (yes/no).

The physical performance was evaluated through questions about the practice of regular physical activity, defined by exercising for at least 150 min per week (yes/no), an estimate of how many kilometers the participants walked weekly, and participation in previous walks.

**Fig. 1:**
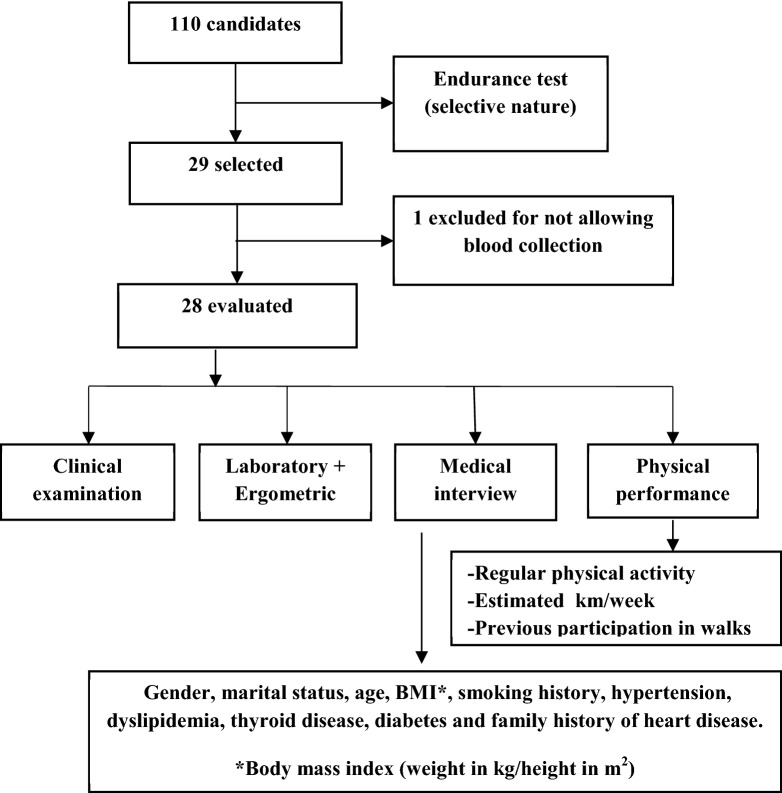
Flowchart of patient selection for the ecological walk of Goiás

All of the above information was self-reported by the athletes (Fig. 1).

### Study design

A longitudinal study was conducted in July 2015 that evaluated 28 race walkers, including 24 men and 4 women, who participated in the “Ecological Walk of Goiás—2015” [7, 8]. The participants walked 310 km in 5 days (average 62 km/day), consisting of alternating periods of fast walking and running, at an average speed of 7.6 km/h.

During the walk, which was characterized by wide variability in temperature and air humidity (16 to 33 °C and 21 to 76%, respectively), water and electrolytes were offered every hour to keep participants hydrated, and snacks, as advised by the Nutrition team, were consumed at an average frequency of every 2 h. In the middle of the day, a break of approximately 2 h was given for lunch, rest, and dressing, according to each walker’s needs. An ambulance with a healthcare team followed the walkers throughout the entire walk (5 days).

Blood samples were collected during the initial interview, in the morning while fasting, and at the end of each day of the walk, at approximately 7 pm, 5 to 60 min after finishing the activity, with the exception of the last day, when no blood was collected for logistical reasons.

Blood was collected through peripheral venous access by a team trained in that technique. For each collection, approximately 5 mL of blood was extracted. One drop of the blood was applied on a blood glucose test strip of the Accu-chek active glucometer (Roche) for determination of capillary blood glucose, and the remaining sample was centrifuged at the collection site at 3000 rpm for 10 min to obtain serum/plasma. Samples were immediately stored at − 20 °C for further analysis.

### Analytical methods

The hormonal tests were performed using the electrochemiluminescence method in the clinical analysis laboratory of one of the participating universities, supervised by a biomedicine physician, using commercial kits of proven quality, which were registered with the National Sanitary Surveillance Agency (ANVISA), according to the manufacturers’ instructions. The tests were performed on automated and semiautomated equipment according to the specific settings and with quality control. To ensure correct execution of the test, all of the instructions given in the user guide for the analyzer were followed. The reference ranges provided by the manufacturer were insulin: 2.6–24.9 μIU/mL; cortisol (6–10 h): 4.8–19.5 μg/dL and (16–20 h): 2.5–11.9 μg/dL; testosterone: men 20–49 years: 249–836 ng/dL, men ≥ 50 years: 193–740 ng/dL, and women 20–49 years: 8.4–48.1 ng/dL; TSH: 0.27–4.2 μIU/mL; FT4: 0.83–1.70 ng/dL; and T3: 0.80–2.00 ng/mL.

### Ethics

The study was approved by the Research Ethics Committee of the Pontifical Catholic University of Goiás; number 1,107,021. All participants signed an informed consent form.

### Statistical analysis

Statistical analysis was performed using Stata^®^ software version 14 (StataCorporation, College Station, Texas, USA). Continuous variables are presented as means and standard deviations, while categorical variables are presented as absolute and percentage values. The Shapiro–Wilk test was used to determine whether continuous variables were normally distributed. Variables with a non-normal distribution were evaluated using the Wilcoxon Sign test (comparison between measures), and the results are presented as P values, whereas the Mann–Whitney test was used for comparisons between groups (male vs. female). Variables with a normal distribution were evaluated using Student’s T test for paired samples when comparing the measurements (values also presented as P values) and Student’s T test for unpaired samples when comparing groups (male vs. female). Values of P < 0.05 were considered significant.

## Results

### Population studied

The participants had a mean age of 45 years (18–64 years) and mean body mass index of 23.0 k/m^2^ (18.4–28 k/m^2^). All athletes practiced regular exercise, averaging 57.14 km per week. Twenty-five percent (25%) of the walkers evaluated were participating in the walk for the first time, while the remaining three quarters had participated in other years, averaging 5 previous walks (Table 1).

**Table 1:** Number of participants, sex, age, anthropometric data, smoking history, personal and family history

Factor	Women	Men	P
N	4	24	
Age	43.00 (± 3.60)	46.18 (± 10.73)	0.57
Weight (kg)	58.08 (± 4.72)	70.38 (± 9.67)	0.021
Height (m)	1.62 (± 0.06)	1.75 (± 0.07)	0.002
BMI (kg/m^2^)	22.07 (± 1.11)	23.17 (± 2.45)	0.39
Ex-smoker	0.00 (0%)	5.00 (21%)	0.31
Family history of cardiovascular disease	3.00 (75%)	6.00 (25%)	0.047
SAH	1.00 (25%)	0.00 (0%)	0.013
DM	0.00 (0%)	1.00 (4%)	0.68
Dyslipidemia, N (%)	0.00 (0%)	1.00 (4%)	0.68
Thyroid disease	0.00 (0%)	1 (4%)	0.68
Practice of physical activity	4 (100%)	24 (100%)	
km per week, mean	22.50 (± 6.45)	62.92 (± 26.14)	0.005
First ecological walk	2 (50%)	5 (21%)	0.21
Number of previous walks	1.75 (± 2.87)	6.08 (±) 6.54	0.21

Values given as the mean (± SD) or n (%)

*SD* standard deviation, *BMI* body mass index (weight in kg/height in m^2^), *SAH* systemic arterial hypertension, *DM* diabetes mellitus, *DLP* dyslipidemia

Few participants reported associated pathologies. A walker had metformin-controlled type 2 diabetes mellitus (dose not reported). Another was hypertensive and medicated with losartan (dose not reported). A third walker who was diagnosed with hypothyroidism was on stable treatment with levothyroxine at a dose of 50 μg/day. Finally, one individual reported non-specific and non-treatable changes in blood lipid levels (Table 1).

### Weight

Analysis of the weight of the 28 participants, specifically the change in daily weight (weight before-weight after walking), showed a loss of − 1.8% of the total weight on the 1st day; − 0.52% on the 2nd day; − 0.8% on the 3rd day; and a paradoxical gain of + 1% on the final day (Table 2).

**Table 2:** Mean weight variation during the days of the walk (n = 28)

Weight (kg)	Baseline^a^	Day 1	Day 2	Day 3	Day 4	Day 5^a^
Morning	69.05 (± 10.15)	70.28 (± 9.90)	69.52 (± 9.79)	69.74 (± 9.91)	69.00 (± 9.88)	70.02 (± 10.10)
Night	–	69.02 (± 9.56)	69.12 (± 9.95)	69.17 (± 9.88)	69.72 (± 10.01)	–
Change in weight^b^	–	− 1.26	− 0.40	− 0.57	+ 0.72	–
% change	–	− 1.79%	− 0.57%	− 0.82%	+ 1.04	–

Values given as mean (± standard deviation)

^a^Weight measured only in the morning

^b^Change in weight: (morning weight-night weight)

### Hormonal measurements

The baseline hormonal assessment, based on a sample taken before walking in the morning, is presented in Table 3. The means of all values are within the reference range. The walker with a history of hypothyroidism exhibited TSH, FT4, and T3 values within the normal range.

**Table 3:** Mean baseline hormone levels (n = 28), measured in the morning

Factor	Women	Men	P
Cortisol (μg/dL)	13.22 (± 3.14)	16.41 (± 3.96)	0.140
T3 (ng/mL)	1.25 (± 0.20)	1.10 (± 0.17)	0.110
FT4 (ng/dL)	1.25 (± 0.17)	1.24 (± 0.13)	0.910
TSH (μIU/mL)	3.12 (± 1.81)	2.62 (± 1.71)	0.600
Insulin (μIU/mL)	8.15 (± 5.01)	5.70 (± 3.87)	0.270
Testosterone (ng/dL)	10.29 (± 7.66)	513.53 (± 145.91)	< 0.001

Values given as the mean (± SD). P values indicate comparisons between men and women

*SD* standard deviation, *T3* triiodothyronine, *FT4* thyroxine, free form, *TSH* thyroid stimulating hormone

The mean values for hormone and glucose levels and the cortisol/testosterone, FT4/T3, and glucose/insulin ratios collected at the end of each day of the walk are presented in Table 4.

**Table 4:** Daily mean values of hormone and glycaemia collected at the end of each day (n = 28)

Variable	M1 Mean (SD)	M2 Mean (SD)	M3 Mean (SD)	M4 Mean (SD)	P value
Cortisol (μg/dL)	17.46 (± 11.85)	11.88 (± 7.86)	11.17 (± 5.39)	8.39 (± 4.04)	0.001
T^a^ (ng/dL)	270.35 (± 161.76)	267.90 (± 140.00)	222.73 (± 150.09)	254.98 (± 121.34)	0.692
T3 (ng/mL)	1.25 (± 0.20)	1.22 (± 0.20)	1.27 (± 0.18)	1.20 (± 0.17)	0.484
FT4 (ng/dL)	1.31 (± 0.13)	1.26 (± 0.14)	1.23 (± 0.16)	1.14 (± 0.14)	< 0.001
TSH (μIU/mL)	3.96 (± 2.51)	3.53 (± 2.06)	3.28 (± 2.13)	3.24 (± 2.02)	0.201
Glucose (mg/dL)	104.96 (± 22.42)	108.71 (± 17.91)	99.96 (± 16.42)	104.71 (± 13.12)	0.729
Insulin (μIU/mL)	4.91 (± 2.71)	5.76 (± 5.69)	3.72 (± 2.47)	5.39 (± 4.15)	0.297
C/T^a^	0.13 (± 0.16)	0.07 (± 0.06)	0.08 (± 0.06)	0.05 (± 0.04)	0.073
FT4/T3	1.05 (± 0.23)	1.06 (± 0.24)	0.99 (± 0.18)	0.97 (± 0.20)	0.038
G/I	30.46 (± 24.86)	30.04 (± 18.40)	32.87 (± 12.03)	28.45 (± 14.71)	0.764

Value in parentheses: standard deviation. P value for trend

*SD* standard deviation, *T* testosterone, *T3* triiodothyronine, *FT4* free thyroxine, *TSH* thyroid stimulating hormone, *C/T* cortisol/testosterone ratio, *FT4/T3* free thyroxine/triiodothyronine ratio, *G/I* glucose/insulin ratio, M1: Mean values of hormones and glucose on the 1st day of the walk; M2: Mean values of hormones and glucose on the 2nd day of the walk; M3: Mean values of hormones and glucose on the 3rd day of the walk, M4: Mean values of hormones and glucose on the 4th day of the walk

^a^Testosterone and Cortisol/Testosterone ratio analyzed only in men. Q: Test analyzed only in men

The daily measurement of cortisol showed a progressive decrease from the 1st to the 4th day of the walk, which was obvious and significant when the 1st and 4th days were compared.

**Fig. 2:**
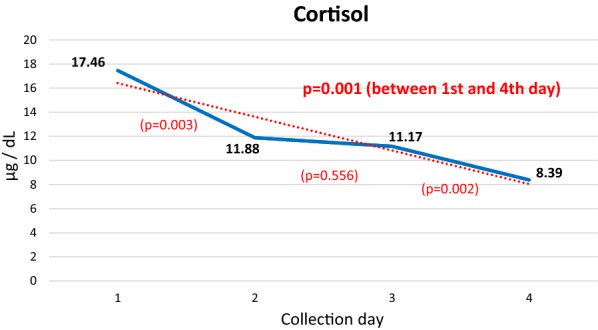
Mean daily cortisol levels. P values between the first and second, second and third, third and fourth, and first and fourth days

The TSH and T3 levels showed little variation during the 5-day walk, with a decreasing trend that was not significant. In contrast, the FT4 level and FT4/T3 ratio showed a significant decreasing trend (Fig. 2).

**Fig. 3:**
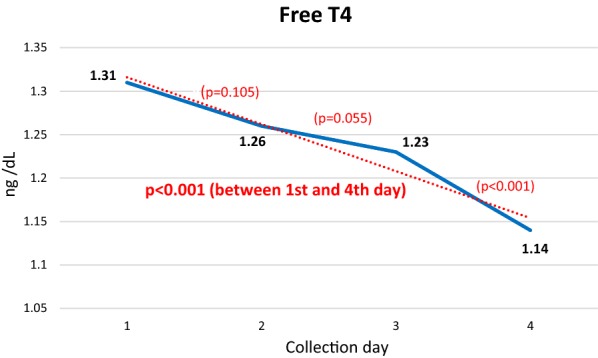
Mean daily FT4 levels. P values between the 1st and 2nd, 2nd and 3rd, 3rd and 4th, and 1st and 4th days

**Fig. 4:**
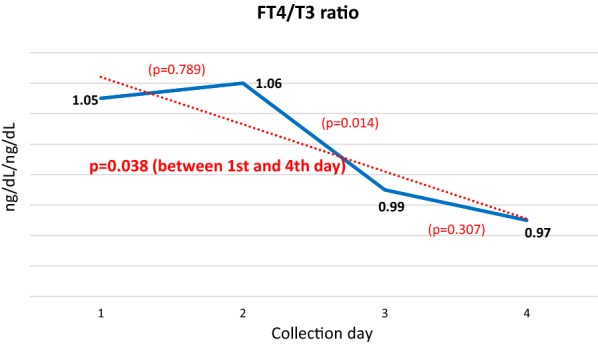
Mean daily FT4/T3 ratio. P values between 1st and 2nd, 2nd and 3rd, 3rd and 4th, and 1st and 4th days

Regarding the testosterone measurements, women were excluded from the sample because of the large discrepancy between the male and female values. Among men, a gradual decreasing trend was noted but with an increase on the last day (Figs. 3 and 4).

Insulin, glycemia, and the glycemia/insulin ratio showed a variable daily behavior with no regular pattern.

## Discussion

Before discussing the hormonal changes themselves, it is important to note that the daily variation in weight during the walk was small, and this minimal change is a consequence of the frequent supply of fluids and food throughout the entire walk, which prevented dehydration and caloric deficits that could interfere with hormonal assessments [5, 9]. An additional aspect that had some influence in our findings is the fact that all participants were well trained amateur athletes, who most certainly had an attenuated hormonal response to exercise when compared to untrained individuals [10].

### Cortisol

Analysis of the variation in cortisol showed a gradual decrease in blood levels throughout the days of the walk, with a significant decreasing trend between the 1st and 4th days. This trend suggests an adaptive process to stress or may indicate that the fatigue induced by the previous exercise may have modified the hormonal response, leading to feedback suppression [11]. According to Kraemer and Rogol [12], a progressive adaptation occurs, with a decreased adrenal response to the adrenocorticotropic hormone (ACTH) released by exercise at the same relative intensity.

In this group of race walkers, the cortisol level measured at the end of the 1st day of walking, collected at night, was surprisingly larger than the baseline value collected in the morning, contrary to expectations based on the circadian rhythm [13, 14]. This increase in cortisol is a consequence of the type of walking exercise, which is considered as medium to high intensity, and the prolonged duration.

The hypothalamic–pituitary–adrenal (HPA) axis is activated during stress. Corticotrophin-releasing hormone (CRH), which has a predominant role, and arginine vasopressin (AVP) play an important role in exercise-induced stimulation of ACTH secretion, which consequently leads to an increase in adrenal cortisol levels [9, 12].

Cortisol is therefore an exercise-responsive hormone that shows a significant increase after high intensity and short duration exercise or submaximal intensity exercise with a longer duration [15]. Therefore, the decrease trend in cortisol response found was not as expected. Although it has been reported, in a study that evaluated cortisol responses to daily strenuous walking during 4 successive days [11], suggesting a progressive adaptive response to stress that could eventually lead to decreasing cortisol values. Since cortisol is a potential biomarker of a catabolic state [16] our findings can be interpreted as beneficial and a chronic effect of this exercise modality.

Thus, two factors modulate the response of the HPA axis to exercise: intensity and duration [9]. The minimum exercise intensity required to produce a cortisol response is 60% of the VO_2_Max. Exercise above this threshold increases the plasma cortisol concentration linearly with exercise intensity [9].

The activation of the HPA axis represents a physiological response to the energetic, metabolic, vascular, neurophysiological, and psychological requirements of exercise [9]. Glucocorticoids have many beneficial effects during physical activity, including increasing the viability of metabolic substrates for the energy needs of the muscle, maintaining normal vascular integrity and responsiveness during exercise, and preventing overactivity of the immune system due to repeated, exercise-induced muscle injury [9].

Independent of thermal stress, hypohydration potentially amplifies the physical activity-induced cortisol response through additional stimulation of AVP and ACTH secretion in a cascade [9]. In this study, this additive increase in cortisol secretion due to dehydration was not relevant because large variations in weight, which would indicate significant losses in body water, were not observed.

### Thyroid hormones

Changes in thyroid hormone levels in response to exercise, in general, are small and within the normal physiological range [17]. The literature shows divergent results regarding the behavior of thyroid hormones in response to exercise [5, 18–20], with reports of an increase, decrease, or no change in the levels of thyroid hormones, regardless of the type of exercise, intensity, and duration. These ambiguous findings are attributed to numerous confounding factors, such as nutritional status and variations in body composition [21, 22].

Interestingly, when the thyroid function was analyzed, the curve of the FT4 levels was similar to that of cortisol: an initial increase on the 1st day, relative to the baseline value, and a subsequent significant decreasing trend (Fig. 3), as if an adaptive reaction of the thyroid occurred in response to the initial stress. Thus, exercise possibly induces an unknown signal to conserve energy that, in a cascade, would lead to decreased secretion of thyroid hormones, unrelated to changes in body composition or degree of hydration [18]. One of the probable mechanisms would be a decrease in leptin levels, which are also affected by decreasing cortisol values, affecting specific receptors in the hypothalamus region that controls TRH expression, with consequent reduced stimulation of thyroid hormone production.

The FT4/T3 ratio showed a significant decreasing trend when the 1st and 4th days of the walk were compared (Fig. 4).

The behavior of the FT4/T3 ratio was opposite that of the metabolism of thyroid hormones during fasting and in other stress situations, i.e., reverse T3 (rT3) increases and T3 decreases as a result of the decrease in 5′-monodeiodination, generating an increase in the FT4/T3 ratio [23, 24]. This protective physiological mechanism, which inhibits the exhaustion of the body’s energy reserves and enables the synthesis of new energy sources [17], was likely not triggered because the food supply was practically continuous and ad libitum during the walk, thus preventing an energy deficit.

### Testosterone

The effect of physical exercise on testosterone levels is also variable in the literature, with contradictory results [4, 25, 26]. There are reports of reduction, increase, and even no change in testosterone concentrations after physical activity. These divergent results may be a consequence of differences in individual physical condition and exercise duration/intensity [25]. In this study, a decrease in testosterone levels greater than 52% was observed relative to the baseline values collected in the morning, which is due in part to the circadian rhythm [27] but also to the effect of exhaustive and long-duration exercise, exceeding 8 h. The slight increase on the last day of the walk can also be attributed to an adaptive effect of the axis to the effort expended.

The cortisol/testosterone ratio decreased significantly from the first to the 2nd day of the walk, remaining at these lower levels on the following days. This pattern represents a greater decrease in cortisol levels than in testosterone concentrations, with the latter remaining at more stable levels with small variations. This fact indicates a more anabolic hormonal environment, which is opposite of the effect observed for more prolonged activities [4]. Again, the constant supply of food during the walk likely reduced the need for amino acid recruitment for gluconeogenesis, favoring a less catabolic profile [4].

The exclusion of women in the analysis of testosterone levels is due to evidence that the levels of this androgen are very low in females, which is different from the characteristic levels of males, thus generating less reliability in the values measured [28].

It is important to note that the only patient with diabetes showed a significant increase in blood glucose levels at the end of the 1st day of the walk but returned to lower values, considered normal, without adjustment of the dose of the hypoglycemic medication. This finding demonstrates the hyperglycemic effect of stress, largely influenced by an increase in cortisol levels, which was neutralized by the reduction of the levels of this glucocorticoid observed during the walk through the adaptive mechanism already mentioned.

One limitation of this study is that blood was collected exclusively at night (except for the baseline collection), when several hormones exhibit reduced levels due to the circadian rhythm, especially cortisol and testosterone. Other limitations related to the study design such as the fact that it was and observational study conducted without control group need to be acknowledged. Additionally, small number of individuals assessed and underrepresentation of female sex are some sample related aspects that can also be considered limitations.

However, a unique and positive feature of this study was that it was performed outside the laboratory, in the real world, without any control of environmental conditions and with high variations in temperature and humidity, people from the community, amateur athletes with a mean age greater than 40 years, and sequential assessment of a hormonal panel of great importance for the practice of physical activity.

The attenuated hormonal response to long-term moderate-to-high intensity exercise found indicate that this kind of activity is safe and harmless. The catabolic effects were lower than expected, as long as adequate nutritional and fluid replacement is provided during the physical activity.

## Conclusion

Medium to high intensity, long-duration, aerobic physical activity for several consecutive days produces small changes, which are often not significant, in the values of the hormones tested but reinforces the hypothesis of an adaptive hormonal mechanism to stress that is particularly notable in the adrenocorticotrophic and thyroid axes.

## Abbreviations

T4: thyroxine
FT4: free thyroxine
T3: triiodothyronine
TSH: thyrotropin/thyroid stimulator hormone
SD: standard deviation
BMI: body mass index
SAH: systemic arterial hypertension
DM: diabetes mellitus
DLP: dyslipidemia
T: testosterone
C: cortisol
G: glycemia
I: insulin
